# Imageomics defines granular morphological changes of human skin with age and reveals a rejuvenating effect of xenografting

**DOI:** 10.64898/2026.04.29.721704

**Published:** 2026-05-04

**Authors:** Austin E. Y. T. Lefebvre, Ying Zheng, Ruifeng Yang, Fiona Lan, Arben Nace, Emilie Katz, Sergiy Libert, Cynthia Kenyon, Katie Podshivalova, George Cotsarelis

**Affiliations:** 1 Calico Life Sciences LLC; 2 University of Pennsylvania; 3 University of Pittsburgh

## Abstract

Rejuvenating aging human skin is a major therapeutic goal, but objective, quantitative measures of intrinsic aging are limited. We performed a cross-sectional histological study of UV-protected buttock and abdominal skin in adults spanning multiple decades of life to identify features that reliably track age. Epidermal thickness measured between rete ridges was unchanged, but rete ridge size declined linearly with age: ridges became shorter and thinner in both sites, though rete ridge number decreased only in the abdomen. Consistent with these structural changes, proliferative cells (Ki67+) per ridge and expression of integrin β4 (ITGB4), a putative stem-cell marker, were reduced in aged skin. We combined these biomarkers into a predictive model that estimated skin age more accurately than any single marker. To test whether the model detects longitudinal change, we analyzed aged abdominal skin before and after xenografting onto young or aged mice, a procedure previously reported to rejuvenate human skin in young but not aged recipient mice. Both individual biomarkers and the imaging model indicated rejuvenation regardless of host age; however, notably, engraftment efficiency was lower in aged hosts, with surviving grafts showing younger histological phenotypes. These results provide quantitative criteria for assessing intrinsic skin aging and suggest that the process of engraftment itself is sufficient to induce rejuvenation-like changes.

## Introduction

The appearance of the skin is perhaps the most overt indicator of one’s age. Aging of the skin has been divided into “intrinsic” and “extrinsic”. Extrinsic is thought to be caused primarily by UV light whereas intrinsic is due to innate or endogenous biological processes, leading to recognized histologic and cellular changes in the skin (Quan 2023; Zhang and Duan 2018). In the aged, protected epidermis, the most pronounced histologic change is flattening of the epidermis from loss of rete ridges often referred to as “epidermal thinning” (Costello et al. 2023; Lavker et al. 1987; Quan 2023; Russell-Goldman and Murphy 2020).

The proportion of Ki-67 positive basal keratinocytes declines with age, presumably reflecting a loss of epidermal stem cells (Costello et al. 2023; Giangreco et al. 2008; Grove and Kligman 1983). Diminished proliferative capacity of basal keratinocytes is thought to contribute to “epidermal thinning” and age-related loss of rete ridges (Giangreco et al. 2008; Rübe et al. 2021; Zou et al. 2021). Chronologic aging correlates not only with reduction in the number of rete ridges but also manifests in discernible structural changes in residual rete ridges, which become shorter and thinner (Costello et al. 2023; Lavker et al. 1987). As the number of rete ridges diminishes, the dermal-epidermal junction flattens with age, and aged skin exhibits reduced expression of the epidermal adhesion molecule ITGB4 (Balin 1989; Langton et al. 2016; Lavker et al. 1987; Lavker 1979). Together, these histologic and molecular changes are indicative of age-associated remodeling of the dermal-epidermal junction, and compromised attachment between the epidermal and dermal compartments.

The dermis also expresses several intrinsic age-related changes. Pruning of the microvasculature of the dermis has been reported to occur with age (Balin 1989; Gunin et al. 2014), as indicated by reduced histological staining for various endothelial cell markers, including CD31 (Arnal-Forné et al. 2024; Gunin et al. 2014). This reduced capillary density presumably affects nutrient and oxygen delivery – regression of vascular loops in the dermal papillae limits available surface area for exchange (Gilchrest 1982). Intrinsic aging also results in degradation and loss of collagen and elastin fibers, with associated remodeling and disorganization of the dermal extracellular matrix (ECM) (Marcos-Garcés et al. 2014; Mora Huertas et al. 2016; Shin et al. 2019; Varani et al. 2006). Changes in the macromolecular structure of the remaining fibers culminate in an increase in dermal stiffness (He et al. 2023; Jiang et al. 2025; Roig-Rosello et al. 2024).

The study of intrinsic aging in skin is complicated by several factors, including UV exposure and differences across anatomical sites. UV radiation arrests proliferation in the interfollicular epidermal stem cell population (Panich et al. 2016) and upregulates expression of matrix metalloproteinases which degrade collagen, elastin, laminin, and other hemidesmosome proteins that anchor the dermal-epidermal junction (Doleckova et al. 2024; Feng et al. 2024). In addition, the morphology of human skin differs across anatomic sites, so conclusions drawn from single-site studies are not necessarily generalizable (Balin 1989).

While any observed morphologic change or biomarker discussed above might individually allow for estimation of chronologic age, consideration of a combination of markers would be more comprehensive and might allow for a more accurate estimation of skin age and health. In addition, these age-dependent biomarkers have not yet been applied to evaluating longitudinal changes in skin integrity, particularly in response to conditions thought to induce rejuvenation or protection from disease states. Prior work has reported that, in a xenograft model, aged human skin grafted onto young immunodeficient host mice exhibits signs of rejuvenation with respect to a series of key biomarkers associated with intrinsic skin aging (Keren et al. 2022). In demonstrating that rejuvenation of aged human skin is possible, this work raises the additional question of how to quantify intrinsic skin aging – an inherently complex and variable phenotype. Such quantification would permit not only a systematic characterization of skin age, but also a standardized means of evaluating the skin’s response to rejuvenation therapies.

Despite the general agreement on the major histologic hallmarks of intrinsic skin aging, most studies have operationalized these phenotypes through manual measurements and semi-quantitative scoring applied to a limited number of representative fields of view, an approach that is time-intensive, can be subjective, and is vulnerable to both inter-observer variability and bias introduced by field selection in a spatially heterogeneous tissue such as skin (Balin and Pratt 1989; Bencze et al. 2021; Brown 2017; Knoblaugh and Himmel 2019). More recently, whole-slide imaging and digital pathology have made it practical to interrogate substantially larger tissue areas on routine H&E sections, enabling standardized, reproducible morphometric quantification (e.g., epidermal thickness distributions, rete ridge density and geometry, and compartmental area fractions) rather than relying on small, manually selected regions (Aeffner et al. 2019; Kumar et al. 2020). In skin specifically, digital approaches can yield more reliable and accurate measurements of dermal and epidermal thickness than traditional methods, supporting a shift toward quantitative readouts as primary endpoints (Turin et al. 2018). Parallel advances in open, scalable software ecosystems for whole-slide analysis (e.g., QuPath) have lowered the barrier to batch processing and objective feature extraction across cohorts, helping to standardize analyses and reduce dependence on manual workflows (Bankhead et al. 2017). Together, these developments suggest that integrating broad-area, image-derived morphometrics from H&E with established aging-associated biomarkers could provide a more comprehensive and less biased framework for estimating “skin age” and for measuring longitudinal change, but baseline distributions across defined age bands in UV-protected, anatomically consistent human skin–and principled methods to combine multiple markers into a predictive model–remain incompletely established.

In this study, we sought to undertake an unbiased identification of age-related changes in histological skin features coupled with a rigorous quantification of established biomarkers of aging in UV-protected skin from two different anatomical sites with the goal of establishing a baseline for each biomarker across designated age bands, and then integrating these individual biomarker parameters into a novel, predictive model of skin age that might more accurately characterize skin health and aging. We then tested this novel model in a previously described xenograft system in order to assess with greater precision the extent of skin rejuvenation. Surprisingly, we found that aged human skin appeared rejuvenated after grafting onto young or old mice.

## Results

### Morphological epidermal changes with age

**Figure 1.**
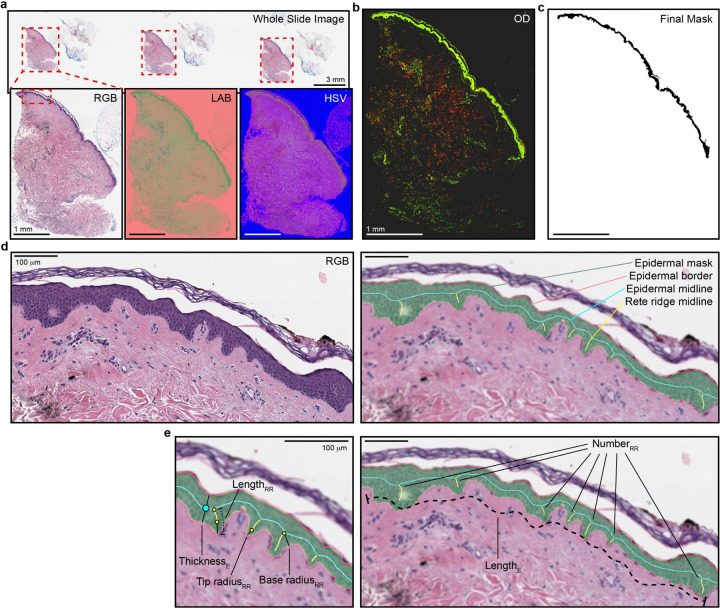
Epidermal segmentation and feature extraction a. Whole slide images of human skin tissue with detected slices’ bounding boxes (red dotted boxes). Images are converted from red-green-blue (RGB) to lightness-a-b (LAB) and hue-saturation-value (HSV) colorspaces. b. Optical density (OD) transformed image obtained via stain deconvolution following RGB, LAB, and HSV derived pre-processing and masking. c. The final epidermal semantic segmentation mask derived from the OD image. d. A close up of the epidermis (left) and automatically acquired segmentations (right) of the epidermal mask, midline, border, and rete ridge midlines. e. A subset of automatically extracted features from rete ridge (RR) and epidermal (E) segmentations. Scale bars represent 100 **microns.**

**Figure 2.**
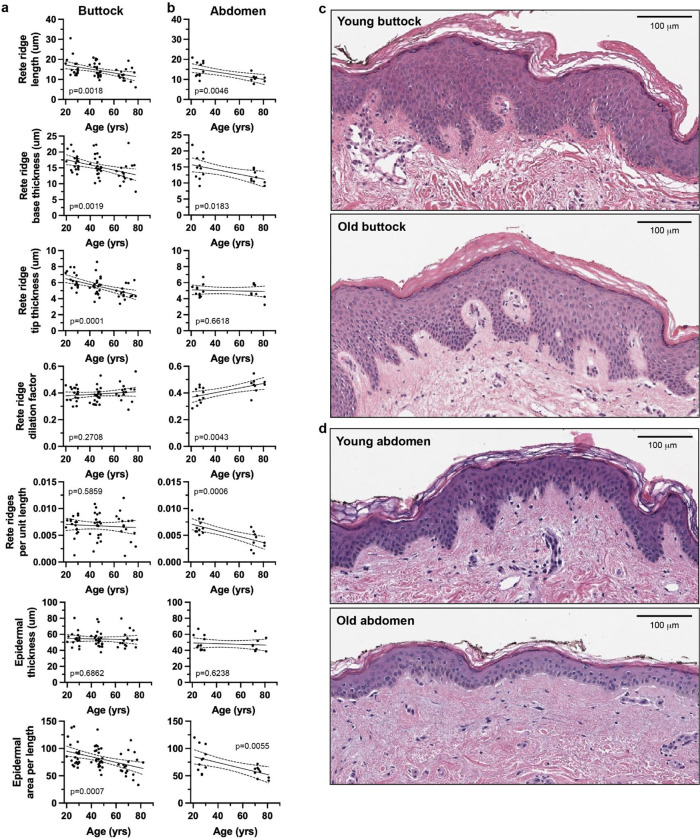
Rete ridge morphology changes with age a. Quantitative epidermal metrics of human buttock skin as a function of age. b. Quantitative epidermal metrics of human abdominal skin as a function of age. c. Representative images of epidermal buttock skin H&E histological images in young (top) and old (bottom) humans. d. Representative images of epidermal abdominal skin H&E histological images in young (top) and old (bottom) humans. n=54 (buttock), n=20 (abdomen). p-value derived via Wald test. Lines represent linear regression model fit +/− 95% confidence interval.

Epidermal features were extracted from digitized H&E-stained skin sections sampled from the buttock (ages 20–30, 40–50, 60–70, and 70+) and abdomen (ages 20–30 and 70+; Supplemental Fig. 1), all from Caucasian female individuals, using a fully automated image processing pipeline. In brief, whole-slide RGB images were downsampled to identify tissue regions, which were segmented into individual slices based on connected components (Fig. 1 a). Each slice was processed at high resolution to isolate the epidermis using color filtering, optical density deconvolution, and morphological operations (Fig. 1b,c). Epidermal regions were skeletonized and converted into graph representations to identify the epidermal midline and rete ridges (Fig. 1d). Morphometric features–including thickness profiles, ridge lengths, and dilation factors–were extracted from each valid region and compiled for quantitative analysis (Fig. 1e). A detailed description of the image-processing methodology is described in the methods section. Linear regression models were independently fit for both buttock (Fig. 2a,c) and abdomen (Fig. 2b,d) samples to assess age-associated trends in morphometric features of the epidermis, specifically focusing on metrics derived from rete ridge geometry and epidermal thickness. All regression model results are presented in full in the supplemental information (Supplemental Table 1).

Across both anatomical sites, no significant age-associated changes were detected in inter-rete ridge epidermal thickness, however there was a significant age-associated decrease in overall epidermal area per length in both buttock and abdominal tissues. Additionally, several morphological features of the rete ridges demonstrated statistically significant linear relationships with age. In general, the length of rete ridges decreased in both abdomen and buttock tissue. Rete ridge base thickness also declined with age across both anatomical locations. Notably, the interquartile range (IQR) of base thickness decreased in both sites, indicating reduced heterogeneity in ridge base dimensions with increasing age. Rete ridge end thickness followed similar trends, suggesting a consistent thinning of the rete end profile with age, particularly in the buttock skin. The ratio of rete ridge end thickness to base thickness (dilation factor) showed a different pattern. In the abdomen, the median dilation factor increased with age, indicating a progressive flattening of rete ridges. These findings suggest that rete ridges become not only shorter but also relatively less sharp with age in abdominal, but not buttock skin. Lastly, the number of rete ridges normalized by epidermal length was the strongest negatively correlated age-association metric in the abdomen, suggesting a marked reduction in rete ridge density in aged abdominal skin. In contrast, surprisingly, the normalized number of rete ridges remained constant with age in the buttock skin.

Collectively, these results demonstrate that while overall epidermal thickness remains stable with age in UV-protected skin, rete ridge morphology–including length, thickness, and density–undergoes consistent and measurable degeneration, with the abdomen showing more pronounced and coherent age-associated trends than the buttock, highlighting the differential susceptibility of skin from different anatomical locations to age-related changes.

### Changes at the epidermal-dermal interface with age

To test whether age-related flattening of rete ridges is mirrored by molecular and vascular remodeling, we quantified basal adhesion, proliferation, and papillary microvasculature in sun-protected buttock skin from the same age-stratified female cohort analyzed above. We measured integrin beta-4 (ITGB4) intensity in the basal epidermis to index hemidesmosomal attachment, counted Ki67-positive nuclei to estimate basal proliferative output, and segmented CD31-positive objects in the papillary dermis to capture microvascular architecture. Immunofluorescence sections were compartment-annotated, features were summarized for each slice, and associations with donor age were tested with linear models. All IF-derived features are summarized in Supplemental Table 2.

#### ITGB4 Expression in the Epidermis Decreases with Age

**Figure 3.**
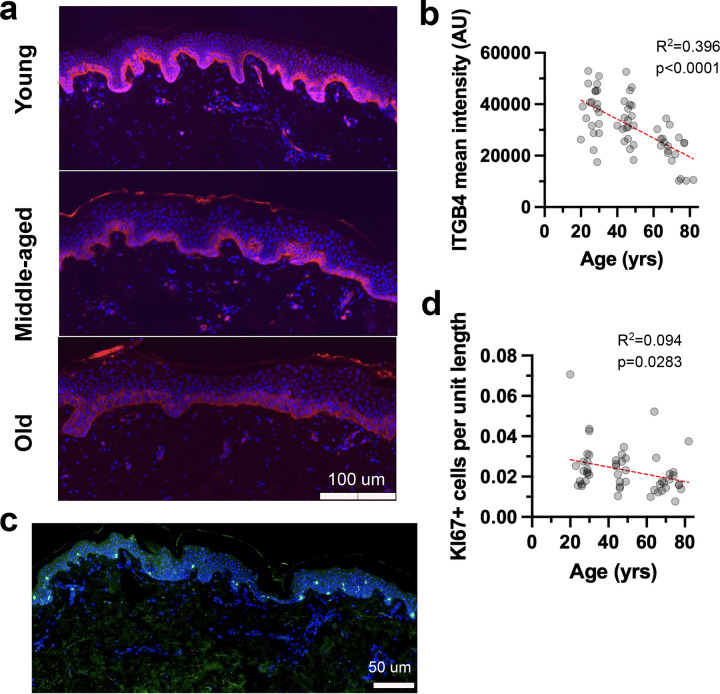
Age-related molecular changes in the basal epidermal layer. a. Representative images of ITGB4 staining (pink) in the epidermal layers of human skin at different ages. ITGB4 staining highlights the basement membrane zone. b. Quantification of ITGB4 staining intensity in the epidermis across age groups. c. Representative immunostaining of MKI67 (green) in human skin. d. Quantification of MKI67-positive epidermal cells across age groups. Mean fluorescence intensity was measured using Fiji analysis software. A simple linear regression model was used to calculate the R squared and p-value (b, d). Data are presented as fit line +/− 95% confidence interval.

Integrin β4 (ITGB4) is specifically expressed in the basal epidermis. It is a component of hemidesmosomes, playing a crucial role for attaching the epidermis to the basement membrane. To assess if ITGB4 expression diminishes with age, contributing to the weakened epidermal-dermal junction observed in aging skin, we quantified its expression in the epidermis across the age-stratified cohort (Fig. 3a). A linear regression model was fit to the mean ITGB4 intensity values across age. The analysis revealed a statistically significant, negative association between age and ITGB4 expression (Fig. 3b).

#### Ki67 Proliferation in the Epidermis Decreases with Age

To evaluate age-related changes in epidermal proliferation, we quantified the number of Ki67-positive cells within manually segmented epidermal regions from buttock skin sections across the age-stratified cohort (Fig. 3c). Linear regression analysis revealed a significant negative association between age and epidermal proliferation in the rete ridges (Fig. 3d). These findings suggest a reduction in the proliferative capacity of the epidermis over time, particularly in the rete ridges, consistent with decreased tissue renewal and turnover observed in aged skin and the diminished volume of the rete ridges in the aged skin.

#### CD31>+ Microvasculature Compaction in the Papillary Dermis with Age

To evaluate whether microvascular characteristics of the papillary dermis change with age, CD31-positive objects were segmented from immunostained images of the buttock skin from the age-stratified cohort

**Figure 4.**
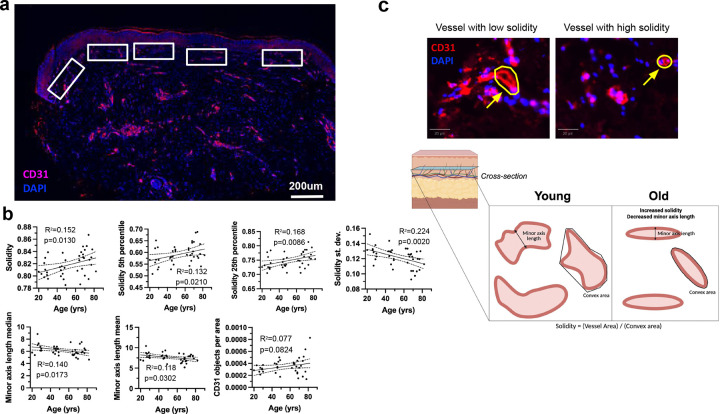
Changes in papillary capillary morphology with age. a. Representative images of CD31 staining for endothelial cells in the papillary dermis. For quantification, five rectangular regions of interest were selected across the papillary dermis in each stained section. b. A simple linear regression model was used to calculate the R squared and p-value. Solidity was calculated for each vessel object as the object’s area over the object’s convex area. Data are presented as fit line +/− 95% confidence interval. c. Model of young and old skin papillary capillary morphometrics.

The quantified features included the fraction of papillary dermis occupied by CD31-positive signal, as well as distributional statistics (mean, standard deviation, 5th, 25th, 50th, 75th, and 95th percentiles, range, and IQR) for a range of geometric and intensity-based object properties (Fig. 4a). These included object area, eccentricity, solidity, perimeter, major and minor axis lengths, aspect ratio, and integrated intensity values (sum, mean, and median intensity). Each of these features was analyzed using linear regression to assess whether there was a significant association with donor age (Supplemental Table 2).

Only two vascular object features showed significant age-associated trends. CD31 objects increased in solidity with age, and the minor axis length decreased with age (Fig. 4b). Surprisingly, no changes in CD31 object density were observed with age. Together these results indicate that papillary dermis microvasculature becomes progressively more compact and morphologically simplified with age (Fig. 4c). The increase in vessel solidity suggests reduced branching or more filled vessel cross-sections, consistent with remodeling or thickening of vascular walls. The concurrent decrease in minor axis length indicates narrowing of individual vascular profiles. These changes imply age-related microvascular consolidation within the papillary dermis, potentially reflecting diminished capillary density and altered perfusion architecture accompanying dermal aging.

In summary, across the age-stratified buttock cohort, epidermal adhesion and proliferation declined and papillary microvascular geometry compacted. ITGB4 intensity in the basal epidermis decreased with age, Ki67-positive cell density in rete ridges was reduced, and CD31-based morphometrics shifted toward higher solidity and shorter minor axes. Linear models confirmed these associations across sections. Together, these analyses indicate coordinated declines in basal adhesion and proliferation alongside compaction of papillary microvessels, linking epidermal thinning to molecular and vascular remodeling with age.

### Predictive Modeling of Skin Age

**Figure 5.**
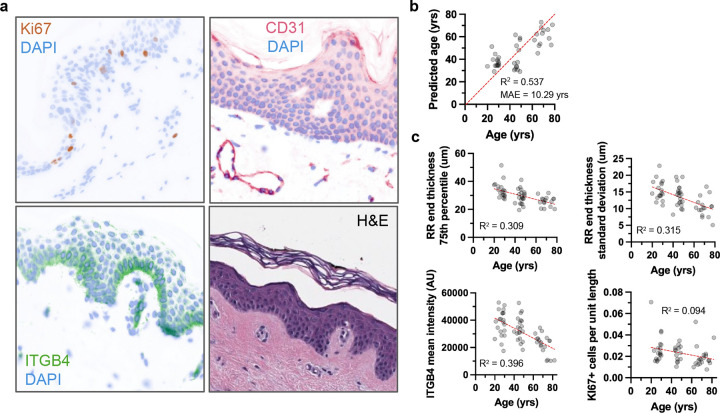
Skin age predictive modeling a. Representative images of Ki67, CD31, and ITGB4 immunohistochemical labeling, and H&E staining. b. Actual versus predicted age as determined by the reduced polynomial random forest regression model. c. Examples of highly age-predicting features used in the random forest model.

We assessed whether histomorphometric and basal molecular features predict chronological age in sun-protected buttock skin via a Random Forest regressor (Fig. 5a). Our model performed better than any individual variable alone, with a mean absolute error (MAE) of 10.29 years with R^2^=0.537 (Fig. 5b).

Feature importance concentrated on rete-ridge end geometry (Supplementary Fig. 3b). The 75th percentile of end-thickness was the single most informative predictor, followed by its standard deviation and range; together these three metrics accounted for ~40% of total importance. Additional contributors included ITGB4 mean intensity, Ki67-positive cell density, and other rete-related thickness metrics, particularly those describing end morphology (Fig. 5c).

### Predictive modeling shows rejuvenation of human skin xenografted into mice

Next, we wanted to test whether the predictive model could be applied to quantify the effect of an anti-aging intervention. It has been previously reported that transplanting aged human skin onto young immunodeficient mice morphologically rejuvenates the xenograft, whereas transplanting the same skin onto old mice has no such effect (Keren et al. 2022).

#### Xenograft survival is impaired in old host mice

**Figure 6.**
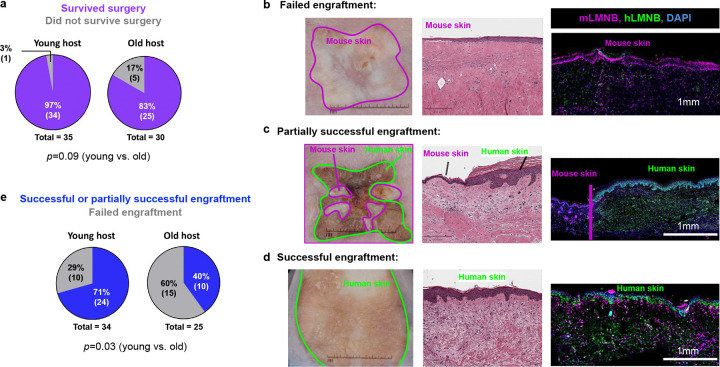
Human skin engraftment is impaired in old mice. Spit-thickness human abdominal skin grafts were transplanted onto immunodeficient nude male mice (nu/nu). a. Proportion of surviving animals after xenografting surgery. b. Example of a failed human skin xenograft. c. Example of partially successful human skin engraftment. d. Example of fully successful human skin engraftment. e. Proportion of successfully engrafted and failed xenografts in young and old mouse hosts. Grafts were examined 2 months following surgery. Representative graft photographs, H&E stains and immunofluorescent species-specific LMNB stains are shown. P-values were calculated using Fisher’s exact test.

To evaluate whether the age of the host environment influences the epidermal age of the skin graft, adjacent samples from the same surgical specimen of abdominal skin from an elderly female donor were xenografted onto both young and old immunodeficient mice. Acute mortality trended higher when the surgery was performed on old animals (17%) as compared to young animals (3%), although the difference was not statistically significant (Fig. 6a). Within the surviving animals, we examined the engraftment success rate. Some xenografts clearly failed to establish, as evidenced by gross morphology and H&E staining, showing presence of only mouse skin at the surgical site (Fig. 6b). To distinguish definitively between human and mouse skin, we used species-specific lamin staining. Whereas in successful xenografts, both human and mouse cells were detected within the graft (Supplemental Fig. 4), in failed xenografts (Supplementary Fig. 5), no human cells were detected, including in the areas with thick epidermis, which could be mistaken for human skin by H&E staining alone. Some grafts had areas of infiltrating mouse skin mixed with the grafted human skin (Fig. 6c) and these were deemed partially successful. Xenografts with only human skin present (Fig. 6d) were deemed fully successful. Overall, the proportion of failed xenografts was significantly higher in old animals (70%) than in young animals (40%; Fig. 6e). For all further histological analyses, only partially or fully successful xenografts were included after excluding any infiltrating mouse tissue.

#### Xenografting Leads to Epidermal Restructuring

**Figure 7.**
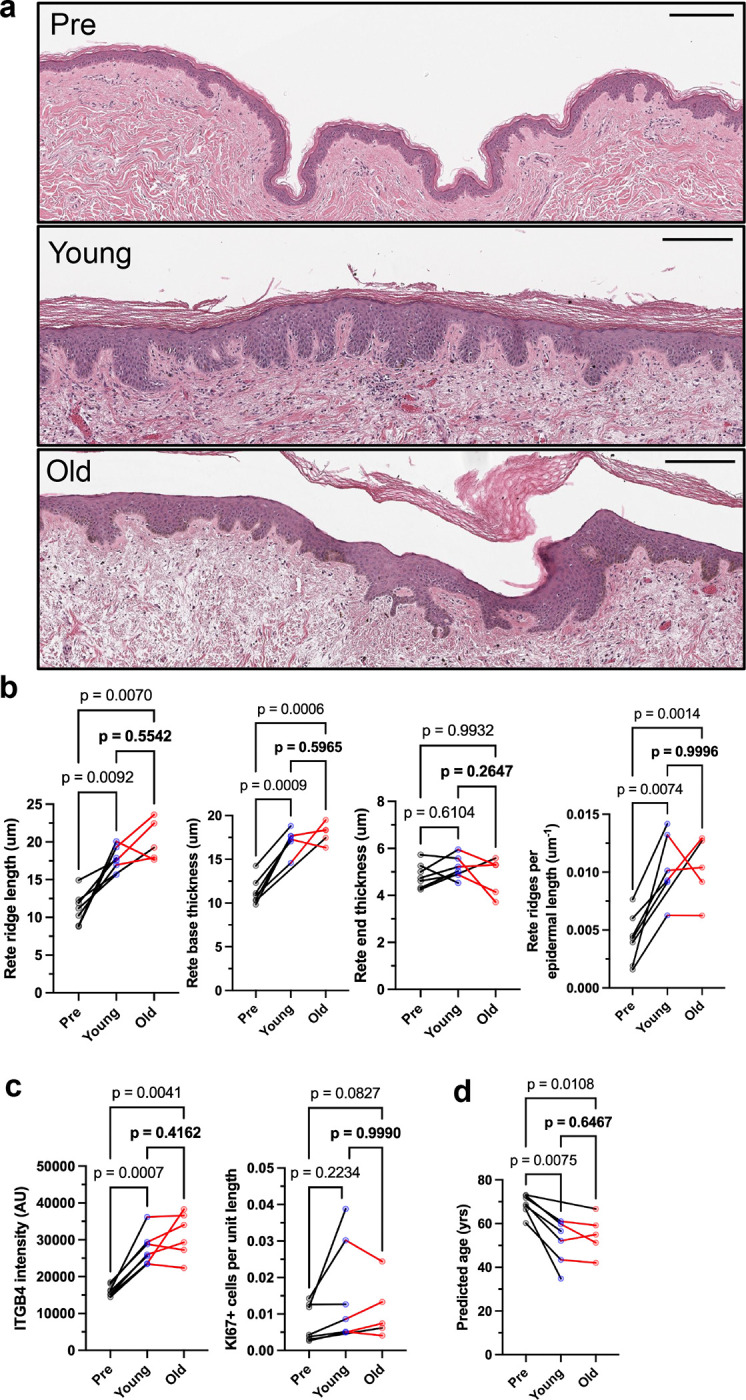
Rejuvenation of successful xenografts occurs independently of host age a. Representative images of a pre-xenograft (Pre, top), post-xenograft in a young mouse (Young, middle), and post-xenograft in an old mouse (Old, bottom) H&E histological images. Scale bars are 200 um. b. H&E-derived rete ridge features in matched pre, young, and old xenografts. c. IF-derived features in matched pre, young, and old xenografts. d. Polynomial random forest regression model-derived predicted skin age in matched pre, young, and old xenografts. N=7, 6, 5 donors for Pre, Young, and Old xenografts, respectively. P derived via a mixed-effects analysis with the Geisser-Greenhouse correction, and Tukey’s multiple comparisons test, with individual variances computed for each comparison.

Following 2 months of graft maturation (which we had previously determined was sufficient time to discern whether the grafting was successful; data not shown), H&E sections were analyzed using the same computational epidermal pipeline described above. For each human donor, pre-graft epidermal metrics were longitudinally compared to post-graft values in both young and old mouse hosts (Fig. 7a). In addition, post-graft metrics were directly compared between the two host age groups to assess whether a young systemic environment could modify or reverse age-associated structural changes in the epidermis (Fig. 7b).

Across the dataset, several features demonstrated statistically significant changes when comparing pre-graft to post-graft conditions. Notably, median rete ridge length increased significantly following grafting to both young (1.60x increase) and old mice (1.80x increase), with no significant difference observed between the two post-graft groups. Median rete ridge base thickness also showed consistent, significant increases post-grafting, increasing by 1.52x in young hosts and 1.59x in old hosts, again with no significant difference between the young and old post-graft host groups. Median rete ridge end thickness showed no significant changes across groups. In contrast, the rete ridge dilation factor, defined as the ratio of end to base thickness, significantly decreased following grafting. The median dilation factor was reduced by 0.76x in young hosts and 0.67x in old hosts, indicating a relative thickening of the base compared to the end. Importantly, the density of rete ridges–quantified as the number of ridges per unit of epidermal length–increased markedly following grafting. Both young and old host groups exhibited similar significant increases in ridge density compared to pre-graft values (young: 2.42x; old: 2.40x), with no detectable difference between the young and old post-graft groups. Inter-rete epidermal thickness, in contrast to rete ridge metrics, showed no consistent or statistically significant changes post-grafting (data not shown).

Together, these results indicate that xenografting induces substantial epidermal restructuring that is independent of host age. Both young and old mouse environments produced similar increases in rete ridge length, base thickness, and density, accompanied by a reduction in dilation factor, reflecting a general remodeling toward a more compact and undulated epidermal architecture characteristic of younger human skin.

#### Xenografting Increases ITGB4 but not Ki67 Expression

To determine whether the host environment influences molecular markers of epidermal integrity and proliferation, we quantified ITGB4 fluorescence intensity and Ki67-positive cell counts in xenografted skin derived from aged human donors (Fig. 7c). As in previous analyses, ITGB4 intensity was measured within manually segmented epidermal regions, and Ki67-positive cells were manually counted and normalized by epidermal length. Comparisons were made between pre-graft samples and post-graft tissues transplanted into either young or old mice, as well as between the two post-graft groups.

For ITGB4, significant increases in expression were observed following xenografting. Mean ITGB4 intensity increased by 1.70x after grafting to young hosts and by 1.93x after grafting to old hosts, relative to pre-graft samples. There was no statistically significant difference between the post-graft ITGB4 levels in young versus old host environments, indicating that the restoration of ITGB4 expression was comparable in both groups. These findings suggest that xenografting alone, independent of host age, is sufficient to restore or enhance expression of basal adhesion markers in aged human epidermis. In contrast, changes in Ki67 expression were not significantly different after grafting.

#### Xenografting Leads to Phenotypically Younger Skin

To assess how xenografting affects the predicted biological age of aged human skin, we applied the Random Forest regression model trained on native buttock skin to grafted samples. Predicted ages were obtained for each slice and averaged across conditions for each donor to generate estimates of age for each of the pre-graft states and post-graft states in either young or old mouse hosts. From these values, within-donor differences were calculated to evaluate the effects of xenografting on age predictions.

Across all donors with complete data, xenografting into either young or old mouse hosts resulted in a consistent decrease in predicted age (Fig. 7d). Among the five donors with available data for both pre-graft and young host conditions, the predicted age decreased by an average of 16.66 +/− 7.73 years. For the four donors with both pre-graft and old host data, the predicted age decreased by an average of 14.49 +/− 4.70 years.

When comparing grafts placed into young versus old hosts directly, differences in predicted age were minimal. The mean difference in predicted age between young and old host environments (Young-Old) was small, with an average 2.25 +/− 4.70 years. These differences were not statistically significant, not large in magnitude and did not follow a consistent direction across donors.

Overall, the model identified a substantial reduction in predicted biological age following xenografting, with both young and old host environments supporting this effect. Notably, the absence of a consistent or pronounced difference between young and old hosts indicates that the reduction in predicted age is driven more by the grafting process itself than by exposure to a young systemic environment. These findings are consistent with our analyses showing that key features such as ITGB4 intensity and rete ridge morphology are restored post-grafting in both host conditions.

## Discussion

The incorporation of fully automated, quantitative methods in this study avoids the subjective variability of manual annotation and enables high-throughput analysis across large image datasets. Our approach provides a high-resolution view of the dermal-epidermal interface by incorporating structural graph modeling of rete ridges that is well-suited to detecting subtle morphological shifts occurring during aging.

Using this automated quantification, we show that aging in sun-protected skin is marked by epidermal–dermal remodeling consistent with weakened basal adhesion, decreased basal proliferation, and compaction of papillary microvessels. ITGB4 declines with age, Ki67-positive cell density decreases in rete ridges, and CD31 morphometrics shift toward narrower, more solid vessel profiles. Surprisingly, we did not detect a gross reduction in the number of microvessels in aged skin, as previously reported (Arnal-Forné et al. 2024; Gunin et al. 2014). Our data suggest that changes in microvessel structure are subtle in UV protected skin, though we did observe compaction of papillary microvessels. These changes align with reduced hemidesmosomal attachment, slower renewal, and constrained perfusion. The data are correlative but internally consistent, indicating concerted remodeling of the epidermal–dermal unit with age.

The predictive modeling results demonstrate that quantitative histological and molecular features of human skin contain measurable information about chronological age. Even with a relatively small dataset, the Random Forest model achieved reasonable accuracy, indicating that epidermal structure and basal layer activity encode age-related signatures detectable by computational methods. The predictive model outperformed any individual feature for predicting donor age (R^2^ = 0.537 for the model, Fig. 5b; R2 = 0.396 for ITGB4, the best individual predictor, Fig. 5c). Performance improvements after dimensionality reduction and polynomial expansion suggest that a limited subset of features, when allowed to interact nonlinearly, captures much of the age-related variance across individuals. The strong weighting of rete ridge end-thickness metrics highlights the morphological remodeling of the dermal–epidermal junction as a key determinant of skin aging. Supporting contributions from ITGB4 and Ki67 further indicate that basal adhesion and proliferative capacity jointly inform epidermal age. Together, these findings show that skin aging is reflected in both architectural and cellular features, and that a small, interpretable feature set is sufficient to build predictive models of epidermal age from histological data.

We demonstrate that xenografting aged human skin into immunodeficient mice triggers a broad structural and molecular rejuvenation of the epidermis, reflected by both morphometric remodeling and reductions in model-predicted biological age. The observed thickening and densification of rete ridges, together with enhanced ITGB4 expression, imply restoration of epidermal integrity and basement membrane adhesion typical of younger skin. In contrast to prior reports (Gilhar et al. 1991; Keren et al. 2022) attributing rejuvenation primarily to systemic factors present in young but not old hosts (“young systemic factors”), we clearly show that these rejuvenating changes occurred regardless of whether the graft was hosted by a young or old mouse. Instead, our findings indicate that the principal driver of rejuvenation is the xenografting process itself, which triggers wound healing and requires revascularization and tissue integration rather than exposure to a youthful systemic milieu.

One possible explanation for these contrasting findings is the different combination of molecular features examined in the published work versus in the current study. Another important difference between the Keren et al. and our study is that in the former, histological analysis of the xenografted skin reportedly was performed 1 to 4 weeks after transplantation (Keren et al. 2022). This timepoint likely is too early to gauge whether a xenograft is successfully incorporated into the mouse, as the healing process is still ongoing and the surgical site is often covered by a scab. For this reason, we analyzed xenografted skin 8–14 weeks after surgery – a time when scabs have detached and visual examination can indicate with high reliability whether human skin is present or replaced by mouse skin tissue (Figure 6b–d; confirmed by species-specific IF staining). A failed xenograft (i.e. only scarred mouse tissue is present at the surgical site; Figure 6b) could easily be mistaken for human skin with a thin flat epidermis, resembling aged human skin, if only H&E staining is used and if gross examination of the surgical site is obscured by a scab. Previous studies using xenografted skin (*e.g.* Mascharak et al. 2025) may need closer scrutiny using species-specific cell markers to ensure conclusions were drawn based on human skin rather than remodeled mouse tissue. Given our finding that engraftment of human skin is significantly impaired in aged mice (Figure 6e), it is possible that such failed xenografts could mistakenly be categorized as xenografts that did not undergo rejuvenation in aged mice.

Our results show that local graft remodeling alone (regardless of host age) can markedly reverse age-associated epidermal features, but the mechanism is unclear. One possibility is that the wound healing process induces the release of growth factors that act directly on epidermal cells (e.g. IGF, KGF). Another possibility is that the process of angiogenesis activated during wound healing rejuvenates skin by increasing skin perfusion. This interpretation is supported by recent reports of anti-aging effects of the vascular endothelial growth factor (VEGF) systemically and in the skin directly (Grunewald et al. 2021; Ichijo et al. 2022)

Why graft survival is impaired in old mice is another outstanding question and could also be linked to angiogenesis. Revascularization is critical for graft incorporation (Resto and Deschler 2009) and mouse skin loses microvessels with age (Ichijo et al. 2022), which would result in impaired anastomosis between human microvessels in the graft and the mouse microvessels in the wound bed. This raises the idea that modulation of the VEGF pathway could be used to increase skin graft survival in aged individuals. Interestingly, Keren et al. (Keren et al. 2022) conclude that VEGF is sufficient to induce rejuvenation of aged human skin xenografted into aged mice. However, this result is also consistent with the idea that VEGF treatment during engraftment may have led to an increased proportion of grafts with rejuvenated morphology due to better graft survival.

Overall, the results presented here demonstrate that histological, molecular, and computational perspectives can be unified to reveal and quantify the architecture of epidermal aging and rejuvenation. Automated, feature-based modeling provides a scalable framework for linking structure to function, enabling both discovery of age-related mechanisms and quantitative assessment of interventions. The convergence of morphometric, molecular, and predictive evidence indicates that epidermal aging is not a diffuse decline but a reproducible, multidimensional process—one that can be measured objectively and, under regenerative contexts such as xenografting, at least partially reversed.

This study is limited in scope, as it largely focuses on the epidermis. For example, dermal age-related changes such as collagen structure were not examined. In addition, no functional readouts, such as skin elasticity or barrier function were performed. It would be interesting to examine these and additional features of skin tissue in future analyses.

## Methods

### Human Skin Samples from Different Age Groups

Normal human skin paraffin blocks from deidentified clinical specimens were obtained from the archived tissue bank of the University of Pennsylvania SBDRC core under IRB protocol #808225. Samples were collected from sun-protected anatomical sites (buttock or abdomen) of female donors and categorized into three age groups: young (20–30 years), middle-aged (40–50 years), and old (≥60 years).

### Split-thickness Human Skin Xenografting Surgery and Analysis

All animal experiments were conducted according to the Institutional Animal Care and Use Committee (IACUC)–approved protocols. Discarded, unidentified human abdominal skin was obtained from TenBio. Immunocompromised nu/nuJ mice were purchased from The Jackson Laboratory. Upon receipt, human skin samples were trimmed to remove subcutaneous adipose tissue and cut into 1.5 × 1.5 cm pieces. An equivalent area of mouse skin was excised to create the graft bed. Recipient mice were either young (7–10 weeks) or aged (14–18 months) at the time of grafting.

Each graft was covered with sterile Vaseline gauze and secured with an elastic bandage, which was removed 10 days postoperatively. Grafts were allowed to heal for 6–10 additional weeks before evaluation and harvest. Harvested grafts were fixed in 4% paraformaldehyde (PFA) at 4°C overnight, dehydrated through graded ethanol, and embedded in paraffin.

### Immunofluorescent Staining of Tissue Sections

Paraffin-embedded tissue blocks were sectioned at 5 μm thickness. Sections were baked at 65°C for 2 hours, deparaffinized in xylene, and rehydrated through descending ethanol concentrations to water. Antigen retrieval was performed in citrate buffer (10 mM, pH 6.0) or Tris-EDTA buffer (10 mM Tris, 1 mM EDTA, pH 9.0) using a pressure cooker for 10–20 minutes. Sections were cooled to room temperature and rinsed with PBS.

After blocking with 5% normal donkey serum in PBS containing 0.1% Tween-20 for 1 hour at room temperature, sections were incubated overnight at 4°C with primary antibodies diluted in blocking buffer. Following PBS-Tween washes, sections were incubated with Alexa Fluor-conjugated secondary antibodies for 1 hour at room temperature in the dark. Nuclei were counterstained with DAPI, and slides were mounted with antifade mounting medium.

Images were acquired using a Leica fluorescence microscope (DMR 6B) under identical settings for each marker to ensure quantitative comparability across samples.

### Primary Antibodies

- Anti-hCD31 (PECAM-1): R&D Systems, AF806
- Anti-MKI67: eBioscience^™^, 48-5698-82
- Anti-ITGB4 (Integrin β4): Cell Signaling Technology, 14803S
- Anti-human LAMIN B1: SYSY Antibodies, HS-404 017
- Anti-mouse Lamin B1: SYSY Antibodies, HS-404 003

### Automated Epidermal Segmentation from Whole Slide H&E Images

Whole-slide images (WSIs) were supplied as SVS RGB files containing a pyramidal series. Each image was read using the tifffile library (Gohlke). For computational efficiency, an initial downsampled version of each slide was generated by selecting the appropriate resolution level with an approximate 16x downsampling factor. If no such level existed in the SVS pyramid, a zoom operation was applied via scipy to the closest larger pyramidal level to achieve the desired resolution(Virtanen et al. 2020). The downsampled image was saved for verification.

To identify tissue-containing regions, the RGB image was converted to HSV color space. Two binary masks were generated: one from the hue channel and another from the minimum intensity across channels. These masks were thresholded using Otsu's method and combined via logical conjunction (Otsu 1979). The resulting binary mask was dilated and hole-filled to generate a preliminary tissue mask. Small connected components (<50,000 pixels) were removed. Labeled connected regions were extracted as distinct tissue slices using bounding box coordinates.

Each detected slice at downsampled resolution was mapped to its corresponding region at level 1 resolution (higher fidelity), using calculated scaling factors between the two image levels. These high-resolution slice images and corresponding binary masks were cropped and processed independently.

For each slice, an epidermis mask was generated via color- and density-based processing. First, Gaussian smoothing with a sigma of 2 was applied to each RGB channel to attenuate high frequency noise. The smoothed image was converted to both LAB and HSV color spaces. A series of thresholds were applied to remove background and non-epidermal regions, including filtering based on lightness, alpha value, hue, and saturation. The following conditional thresholds on which to mask were empirically set: L<50; L>140 (abdomen) or L>160 (grafted tissue) or L>200 (buttock); L<100 and A<150; H<110; S<40; R=G=B. The remaining tissue was deconvolved into optical density (OD) channels using a PCA-based stain separation algorithm (Ruifrok and Johnston 2001). The nuclear channel was isolated by retaining OD intensities above an empirically set threshold of 0.2, followed by Gaussian filtering and Otsu thresholding to generate a nuclear density mask. Binary morphological operations (opening, filtering small objects) were applied. The resulting binary mask is further opened (4 iterations) and uniformly filtered (10×10). Pixels with local mean > 0.5 are labelled epidermis.

### Manual Epidermal Area Quantification and Rete Ridge Analysis

For manual epidermal area quantification, H&E-stained images were calibrated in Fiji (ImageJ, NIH, USA) according to the microscope scale bar. A 1 mm linear region of the epidermis was selected for each sample. The epidermal compartment–defined from the basal layer at the dermal-epidermal junction to the upper boundary of the stratum granulosum–was manually outlined using the polygon selection tool. The enclosed area was measured using the Analyze -> Measure function in Fiji.

Rete ridges were counted along the same 1 mm linear region of the epidermis. Each downward projection of the basal layer into the dermis was defined as one rete ridge. The number of rete ridges per millimeter was recorded as the rete ridge density.

### Epidermal Graph Construction and Feature Extraction

Connected components within the epidermis mask were labeled using a connectivity of 8. These components were treated as candidate epidermal regions and referred to as “trees.” Label masks and associated region properties were extracted. A k-d tree of the tissue boundary was constructed to enable fast nearest-neighbor queries for thickness measurements (1975).

Each epidermal tree was skeletonized using morphological thinning in scikit-image (van der Walt et al. 2014; Zhang and Suen 1984). Nodes and branches in the skeleton were defined using a 3×3 convolutional neighborhood kernel where pixels with greater than 2 neighboring pixels are considered junction nodes, and pixels with exactly 1 neighboring pixel are considered endpoints nodes. Skeleton pixels not belonging to nodes are connected-component labelled as branches and associated with the two nodes they connect; only branches with exactly two nodes are retained. Nodes become vertices; branches become weighted edges (weight = branch length). The minimum spanning tree is computed via networkx (Hagberg et al. 2008). A two-pass Dijkstra search is performed to obtain the longest path in the graph (Dijkstra 1959). Paths shorter than 500 pixels are discarded (slice rejected). Edges on the longest path are marked midline; all other edges are provisional rete ridges. For every provisional ridge, the endpoint node centroids were queried against the tissue boundary k-d tree to compute distances. Branches where the distal tip (endpoint) was closer to the boundary than the junction (base) were discarded while remaining branches were retained as valid rete ridges.

Quantitative morphological features were computed in and aggregated in numpy for each slice and written to a cumulative CSV file via pandas (Harris et al. 2020; McKinney 2010). Extracted features included:

- Epidermal Length: Number of skeleton pixels in the midline.
- Epidermal Thickness: For every midline pixel, perpendicular distance to the nearest external tissue boundary is obtained via KD-tree and doubled.
- Rete Ridge Length: Estimated as the sum of the branch length plus the distances from the base to the midline and the tip to the tree boundary.
- Basal and apical rete ridge thickness: Distances from ridge bases and ridge ends to the external epidermal boundary (x2)
- Rete ridge Dilation Factor: Ratio of end thickness to base thickness for each ridge.
- Rete Ridge Count: Total number of validated ridges.
- Rete Ridge Density: Ridge count normalized by epidermal length.

All image processing, feature extraction, and data analysis was done in python 3.13, with the following dependencies: imagecodecs=2025.3.30, networkx=3.5, numpy=2.3.2, opencv-python=4.11.0.86, pandas=2.3.1, scikit-image=0.25.2, scipy=1.16.1, statsmodels=0.14.5, tifffile=2025.6.11

### ITGB4 quantification

Because ITGB4 is specifically expressed in basal keratinocytes, the basal epidermal layer was manually selected from each section, and mean fluorescence intensity was measured using Fiji.

### KI67 quantification

Epidermal proliferation was assessed by manual quantification of MKI67 (Ki67)-positive nuclei within the epidermis of each histological section. A defined epidermal compartment was used for all analyses. To account for regional variation in epidermal size, the total number of MKI67-positive cells per section was normalized to the measured epidermal length, yielding a proliferation index expressed as MKI67-positive cells per unit epidermal length. In addition, MKI67-positive cells located within rete ridges were specifically counted, and this value was normalized to the total number of rete ridges per section to determine the average number of proliferative cells per rete ridge.

### CD31 quantification

CD31 immunofluorescence images were analyzed to quantify microvasculature within the papillary dermis. For each histological section, the papillary dermis was manually annotated to define the region of interest. Within this region, CD31-positive signal was identified using intensity-based thresholding and converted to a binary representation. Connected components analysis was used to segment individual CD31-positive structures. Quantitative features were extracted from segmented objects and aggregated at the section level. In parallel, microvasculature density was calculated by measuring the total CD31-positive area and expressing it as a percentage of the annotated papillary dermal area. For each sample, five papillary dermis regions were analyzed, and values were averaged to obtain a mean microvascular density per sample for comparison across age groups.

### Random Forest Modeling for Age Prediction

To assess whether quantitative histological features could be used to predict chronological age, we trained Random Forest regression models on 41 samples with complete measurements, using 48 quantitative predictors spanning H&E-derived morphometrics, basal ITGB4 intensity, and Ki67-positive cell counts extracted from human buttock skin samples. The dataset included subjects across a wide adult age range, and all samples used for modeling were from female individuals without pathological annotations. We evaluated the models with a five-fold cross validation.

Patient metadata, including age and sample identifier, was first loaded from a structured CSV file. Quantitative features from hematoxylin and eosin (H&E) stained sections, ITGB4 immunofluorescence (IF), and Ki67 IF were separately loaded and joined by a common accession ID. Each of these datasets included precomputed, slice-level aggregated features (e.g., median rete ridge thickness, mean object eccentricity, etc.) previously extracted using standardized segmentation and quantification pipelines. The final dataset consisted of numeric predictors covering a range of biological domains: epidermal morphometrics (thickness and rete ridge features), basal adhesion marker intensity (ITGB4), and proliferation marker counts (Ki67 per unit epidermal length).

We used a Random Forest regressor from the sklearn.ensemble module to predict donor age. Two versions of the model were trained: Full Feature Model - included all available features; Reduced Polynomial Model - included the top 8 features of the full feature model with second degree polynomial features added. The feature matrix X consisted of all numerical predictors excluding Age and ID. The target variable y was the chronological age of the subject in years. Random Forest parameters were set as follows: 500 trees (n_estimators=500). All trees were trained in parallel (n_jobs=−1) with a fixed random seed (random_state=42) for reproducibility. Model performance was evaluated using 5-fold cross-validation via KFold (5 splits, shuffle=True, random_state=42). Two performance metrics were computed: the mean absolute error (MAE) and the coefficient of determination (R^2^). After training, the model was fit to the full dataset and feature importances were extracted based on mean decrease in impurity. The first model achieved a MAE of 13.16 years and R^2^=0.241 (Supplementary Fig. 3a). To compress the feature set while permitting simple nonlinear interactions, predictors were ranked by impurity-based importance, the top eight were retained, and a degree-2 polynomial expansion without bias terms was applied (8 linear, 8 squared, 28 pairwise products; 44 total predictors). The second model achieved a MAE of 10.29 years with R^2^=0.537 across a 5-fold cross-validation.

### Application of Random Forest Age Prediction Model to Xenografted Skin

To evaluate how xenografting affects predicted biological age of skin, we applied the Reduced Polynomial Random Forest Regression Model described above to the grafted human skin dataset.

Quantitative features for the xenograft experiment were extracted from H&E-stained sections, ITGB4 immunofluorescence, and Ki67 immunofluorescence. For each dataset, a unique identifier column was generated by concatenating the donor Sample number with the graft condition Age class (e.g., "Pre", "Young", or "Old"). This allowed for unambiguous merging across datasets and linkage of replicate slices from the same donor and condition. Each dataset was subset to retain only the predefined list of model-relevant features, which matched those used during training of the Random Forest model. The individual feature tables were merged using the ID column to generate a single feature matrix for inference.

To summarize prediction results at the donor level, predicted ages were averaged across slices within each (Sample, AgeClass) pair. The resulting per-sample means were pivoted into a wide-format table with one row per donor and one column for each condition (Pre, Young, and Old). From this table, within-donor differences in predicted age were calculated as:

- delta_Pre-Young: change in predicted age from pre-graft to post-graft in young mouse hosts
- delta_Pre-Old: change from pre-graft to post-graft in old hosts
- delta_Young-Old: difference in predicted age between grafts placed in young versus old hosts

## Supplementary Material

Supplement 1
