# Supplementary material for: Imageomics defines granular morphological changes of human skin with age and reveals a rejuvenating effect of xenografting
Source: bioRxiv. 2026 May 4:2026.04.29.721704. Preprint. [Version 1] doi: 10.64898/2026.04.29.721704 (PMC13174456; doi:10.64898/2026.04.29.721704)
Supplement: Supplement 1 [file NIHPP2026.04.29.721704v1-supplement-1.pdf]

bioRxiv preprint doi: https://doi.org/10.64898/2026.04.29.721704; this version posted May 4, 2026. The copyright holder for this preprint (which was not certified by peer review) is the author/funder, who has granted bioRxiv a license to display the preprint in perpetuity. It is made available under aCC-BY 4.0 International license.

| **Supplementary Table 1. Skin Characterization Sample List** | | | | | | | | | |
|---|---|---|---|---|---|---|---|---|---|
| **Buttock** | | | | | | **Abdomen** | | | |
| **Young** | | **Middle-aged** | | **Old** | | **Young** | | **Old** | |
| Donor | Age | Donor | Age | Donor | Age | Donor | Age | Donor | Age |

bioRxiv preprint doi: https://doi.org/10.64898/2026.04.29.721704; this version posted May 4, 2026. The copyright holder for this preprint (which was not certified by peer review) is the author/funder, who has granted bioRxiv a license to display the preprint in perpetuity. It is made available under aCC-BY 4.0 International license.

| | | | | | | | | | |
|---|---|---|---|---|---|---|---|---|---|
| 1 | 29 | 1 | 47 | 1 | 77 | | 21 | | 81 |
| 2 | 30 | 2 | 46 | 2 | 68 | | 24 | | 81 |
| 3 | 27 | 3 | 49 | 3 | 67 | | 25 | | 74 |
| 4 | 27 | 4 | 46 | 4 | 64 | | 25 | | 73 |
| 5 | 20 | 5 | 44 | 5 | 75 | | 27 | | 72 |
| 6 | 26 | 6 | 44 | 6 | 74 | | 27 | | 72 |
| 7 | 30 | 7 | 49 | 7 | 68 | | 27 | | 72 |
| 8 | 27 | 8 | 48 | 8 | 62 | | 28 | | 70 |
| 9 | 29 | 9 | 45 | 9 | 78 | | 30 | | 70 |
| 10 | 23 | 10 | 49 | 10 | 70 | | 30 | | 70 |
| 11 | 29 | 11 | 44 | 11 | 72 | | 30 | | |
| 12 | 28 | 12 | 46 | 12 | 69 | | | | |
| 13 | 30 | 13 | 47 | 13 | 66 | | | | |
| 14 | 25 | 14 | 44 | 14 | 82 | | | | |
| 15 | 28 | 15 | 48 | 15 | 65 | | | | |
| 16 | 21 | 16 | 47 | 16 | 68 | | | | |
| 17 | 29 | 17 | 45 | 17 | 64 | | | | |
| 18 | 29 | 18 | 46 | 18 | 74 | | | | |

bioRxiv preprint doi: https://doi.org/10.64898/2026.04.29.721704; this version posted May 4, 2026. The copyright holder for this preprint (which was not certified by peer review) is the author/funder, who has granted bioRxiv a license to display the preprint in perpetuity. It is made available under aCC-BY 4.0 International license.

| | | | | | | | | | |
|---|---|---|---|---|---|---|---|---|---|
| 19 | 24 | 19 | 40 | 19 | 72 | | | | |
| 20 | 30 | 20 | 46 | | | | | | |
| 21 | 24 | 21 | 48 | | | | | | |
| | | 22 | 43 | | | | | | |
| **Ave.** | **26.90** | **Ave.** | **45.95** | **Ave.** | **70.26** | **Ave.** | **26.73** | **Ave.** | **73.50** |
| **SDV** | **3.015** | **SDV** | **2.236** | **SDV** | **5.373** | **SDV** | **2.832** | **SDV** | **4.170** |

**Supplementary Table 2.**

**Xenograft Donor List**

| Donor | Age |
|---|---|
| 1 | 66 |
| 2 | 75 |
| 3 | 69 |
| 4 | 71 |
| 5 | 71 |
| 6 | 69 |
| 7 | 69 |

bioRxiv preprint doi: https://doi.org/10.64898/2026.04.29.721704; this version posted May 4, 2026. The copyright holder for this preprint (which was not certified by peer review) is the author/funder, who has granted bioRxiv a license to display the preprint in perpetuity. It is made available under aCC-BY 4.0 International license.

| | |
|---|---|
| **Average** | **70.00** |
| **SDV** | **2.769** |

bioRxiv preprint doi: https://doi.org/10.64898/2026.04.29.721704; this version posted May 4, 2026. The copyright holder for this preprint (which was not certified by peer review) is the author/funder, who has granted bioRxiv a license to display the preprint in perpetuity. It is made available under aCC-BY 4.0 International license.

## Supplemental Tables

### Supplemental Table 1. All H&E-derived features

| site | feature | int | slope | p | r2 | n |
|---|---|---|---|---|---|---|
| Abdomen | epidermal_area_per_length | 96.2100 | -0.5490 | 0.0055 | 0.3563 | 20 |
| buttock | epidermal_area_per_length | 105.4000 | -0.5121 | 0.0007 | 0.1735 | 63 |
| Abdomen | num_rete_ridges | 38.2865 | -0.2401 | 0.1838 | 0.0960 | 20 |
| buttock | num_rete_ridges | 38.8402 | -0.1235 | 0.3636 | 0.0159 | 54 |
| Abdomen | rete_base_thickness_iqr | 8.5241 | -0.0578 | 0.0223 | 0.2578 | 20 |
| buttock | rete_base_thickness_iqr | 8.9112 | -0.0531 | 0.0076 | 0.1292 | 54 |
| Abdomen | rete_base_thickness_max | 35.5740 | -0.1762 | 0.0049 | 0.3629 | 20 |
| buttock | rete_base_thickness_max | 37.5583 | -0.1560 | 0.0163 | 0.1060 | 54 |
| Abdomen | rete_base_thickness_mean | 17.8958 | -0.0780 | 0.0072 | 0.3382 | 20 |
| buttock | rete_base_thickness_mean | 20.0644 | -0.0861 | 0.0015 | 0.1781 | 54 |
| Abdomen | rete_base_thickness_median | 17.2377 | -0.0749 | 0.0183 | 0.2720 | 20 |
| buttock | rete_base_thickness_median | 19.1991 | -0.0819 | 0.0019 | 0.1714 | 54 |
| Abdomen | rete_base_thickness_min | 5.7145 | -0.0110 | 0.5612 | 0.0191 | 20 |
| buttock | rete_base_thickness_min | 7.8766 | -0.0250 | 0.1909 | 0.0327 | 54 |
| Abdomen | rete_base_thickness_q25 | 12.7875 | -0.0391 | 0.0641 | 0.1777 | 20 |
| buttock | rete_base_thickness_q25 | 15.4304 | -0.0629 | 0.0028 | 0.1589 | 54 |
| Abdomen | rete_base_thickness_q75 | 21.5080 | -0.0994 | 0.0094 | 0.3194 | 20 |
| buttock | rete_base_thickness_q75 | 24.1985 | -0.1133 | 0.0010 | 0.1897 | 54 |
| Abdomen | rete_base_thickness_range | 30.1342 | -0.1665 | 0.0127 | 0.2987 | 20 |
| buttock | rete_base_thickness_range | 29.2425 | -0.1199 | 0.0803 | 0.0577 | 54 |
| Abdomen | rete_base_thickness_std | 6.9522 | -0.0386 | 0.0096 | 0.3180 | 20 |
| buttock | rete_base_thickness_std | 6.8642 | -0.0287 | 0.0376 | 0.0805 | 54 |
| Abdomen | rete_dilation_factor_iqr | 0.2989 | -0.0006 | 0.2795 | 0.0646 | 20 |
| buttock | rete_dilation_factor_iqr | 0.2765 | -0.0004 | 0.2104 | 0.0300 | 54 |
| Abdomen | rete_dilation_factor_max | 1.1406 | -0.0008 | 0.8407 | 0.0023 | 20 |
| buttock | rete_dilation_factor_max | 1.1141 | -0.0020 | 0.4080 | 0.0132 | 54 |
| Abdomen | rete_dilation_factor_mean | 0.4013 | 0.0012 | 0.0223 | 0.2577 | 20 |
| buttock | rete_dilation_factor_mean | 0.4242 | 0.0003 | 0.4746 | 0.0099 | 54 |
| Abdomen | rete_dilation_factor_median | 0.3326 | 0.0017 | 0.0043 | 0.3717 | 20 |

bioRxiv preprint doi: https://doi.org/10.64898/2026.04.29.721704; this version posted May 4, 2026. The copyright holder for this preprint (which was not certified by peer review) is the author/funder, who has granted bioRxiv a license to display the preprint in perpetuity. It is made available under aCC-BY 4.0 International license.

| | | | | | | |
|---|---|---|---|---|---|---|
| buttock | rete_dilation_factor_median | 0.3681 | 0.0005 | 0.2708 | 0.0233 | 54 |
| Abdomen | rete_dilation_factor_min | 0.0793 | 0.0015 | 0.0143 | 0.2899 | 20 |
| buttock | rete_dilation_factor_min | 0.1054 | 0.0007 | 0.1328 | 0.0429 | 54 |
| Abdomen | rete_dilation_factor_q25 | 0.2281 | 0.0016 | 0.0079 | 0.3317 | 20 |
| buttock | rete_dilation_factor_q25 | 0.2584 | 0.0005 | 0.2480 | 0.0256 | 54 |
| Abdomen | rete_dilation_factor_q75 | 0.5235 | 0.0010 | 0.2234 | 0.0812 | 20 |
| buttock | rete_dilation_factor_q75 | 0.5371 | 0.0001 | 0.7875 | 0.0014 | 54 |
| Abdomen | rete_dilation_factor_range | 1.0724 | -0.0026 | 0.5528 | 0.0199 | 20 |
| buttock | rete_dilation_factor_range | 1.0010 | -0.0027 | 0.2989 | 0.0207 | 54 |
| Abdomen | rete_dilation_factor_std | 0.2481 | -0.0004 | 0.4864 | 0.0273 | 20 |
| buttock | rete_dilation_factor_std | 0.2337 | -0.0004 | 0.3197 | 0.0190 | 54 |
| Abdomen | rete_end_thickness_iqr | 3.8729 | -0.0110 | 0.3140 | 0.0563 | 20 |
| buttock | rete_end_thickness_iqr | 5.2596 | -0.0326 | 0.0000 | 0.2963 | 54 |
| Abdomen | rete_end_thickness_max | 20.9404 | -0.1225 | 0.0048 | 0.3651 | 20 |
| buttock | rete_end_thickness_max | 24.6322 | -0.1490 | 0.0001 | 0.2458 | 54 |
| Abdomen | rete_end_thickness_mean | 6.6025 | -0.0146 | 0.1149 | 0.1323 | 20 |
| buttock | rete_end_thickness_mean | 8.3592 | -0.0402 | 0.0000 | 0.3035 | 54 |
| Abdomen | rete_end_thickness_median | 5.1750 | -0.0035 | 0.6618 | 0.0109 | 20 |
| buttock | rete_end_thickness_median | 7.2022 | -0.0351 | 0.0001 | 0.2697 | 54 |
| Abdomen | rete_end_thickness_min | 1.8272 | 0.0064 | 0.2473 | 0.0736 | 20 |
| buttock | rete_end_thickness_min | 2.2973 | -0.0023 | 0.6701 | 0.0035 | 54 |
| Abdomen | rete_end_thickness_q25 | 3.8526 | -0.0022 | 0.7021 | 0.0083 | 20 |
| buttock | rete_end_thickness_q25 | 4.8829 | -0.0170 | 0.0061 | 0.1360 | 54 |
| Abdomen | rete_end_thickness_q75 | 7.7457 | -0.0111 | 0.4136 | 0.0375 | 20 |
| buttock | rete_end_thickness_q75 | 10.2812 | -0.0523 | 0.0000 | 0.3155 | 54 |
| Abdomen | rete_end_thickness_range | 19.0560 | -0.1326 | 0.0056 | 0.3551 | 20 |
| buttock | rete_end_thickness_range | 22.4260 | -0.1464 | 0.0003 | 0.2270 | 54 |
| Abdomen | rete_end_thickness_std | 4.3229 | -0.0246 | 0.0106 | 0.3113 | 20 |
| buttock | rete_end_thickness_std | 5.0030 | -0.0311 | 0.0000 | 0.3080 | 54 |
| Abdomen | rete_length_iqr | 9.9859 | -0.0534 | 0.0216 | 0.2600 | 20 |
| buttock | rete_length_iqr | 10.0318 | -0.0436 | 0.1012 | 0.0508 | 54 |
| Abdomen | rete_length_max | 46.6038 | -0.2725 | 0.0245 | 0.2508 | 20 |
| buttock | rete_length_max | 48.3729 | -0.1035 | 0.6409 | 0.0042 | 54 |
| Abdomen | rete_length_mean | 19.9689 | -0.1093 | 0.0030 | 0.3942 | 20 |
| buttock | rete_length_mean | 20.1842 | -0.0753 | 0.0480 | 0.0731 | 54 |
| Abdomen | rete_length_median | 17.5865 | -0.0942 | 0.0046 | 0.3673 | 20 |
| buttock | rete_length_median | 19.1932 | -0.0993 | 0.0018 | 0.1719 | 54 |
| Abdomen | rete_length_min | 7.4284 | -0.0274 | 0.1148 | 0.1324 | 20 |

bioRxiv preprint doi: https://doi.org/10.64898/2026.04.29.721704; this version posted May 4, 2026. The copyright holder for this preprint (which was not certified by peer review) is the author/funder, who has granted bioRxiv a license to display the preprint in perpetuity. It is made available under aCC-BY 4.0 International license.

| | | | | | | |
|---|---|---|---|---|---|---|
| buttock | rete_length_min | 8.2579 | -0.0356 | 0.0151 | 0.1083 | 54 |
| Abdomen | rete_length_q25 | 13.6773 | -0.0748 | 0.0120 | 0.3024 | 20 |
| buttock | rete_length_q25 | 14.7558 | -0.0752 | 0.0007 | 0.1981 | 54 |
| Abdomen | rete_length_q75 | 23.9100 | -0.1323 | 0.0043 | 0.3718 | 20 |
| buttock | rete_length_q75 | 24.6975 | -0.1164 | 0.0074 | 0.1299 | 54 |
| Abdomen | rete_length_range | 38.8403 | -0.2377 | 0.0449 | 0.2052 | 20 |
| buttock | rete_length_range | 40.4046 | -0.0713 | 0.7479 | 0.0020 | 54 |
| Abdomen | rete_length_std | 9.4188 | -0.0537 | 0.0410 | 0.2121 | 20 |
| buttock | rete_length_std | 8.3714 | 0.0021 | 0.9679 | 0.0000 | 54 |
| Abdomen | rete_ridges_per_epidermal_length | 0.0083 | -0.0001 | 0.0006 | 0.4927 | 20 |
| buttock | rete_ridges_per_epidermal_length | 0.0072 | 0.0000 | 0.5859 | 0.0057 | 54 |
| Abdomen | thickness_iqr | 21.0268 | -0.0137 | 0.8836 | 0.0012 | 20 |
| buttock | thickness_iqr | 19.5923 | 0.0541 | 0.5451 | 0.0071 | 54 |
| Abdomen | thickness_max | 140.7351 | -0.1774 | 0.7460 | 0.0060 | 20 |
| buttock | thickness_max | 237.8079 | -1.1637 | 0.2532 | 0.0250 | 54 |
| Abdomen | thickness_mean | 56.5812 | -0.0472 | 0.6603 | 0.0110 | 20 |
| buttock | thickness_mean | 72.2784 | -0.1385 | 0.4032 | 0.0135 | 54 |
| Abdomen | thickness_median | 50.0275 | -0.0426 | 0.6238 | 0.0136 | 20 |
| buttock | thickness_median | 55.5605 | -0.0311 | 0.6862 | 0.0032 | 54 |
| Abdomen | thickness_min | 27.1436 | -0.0138 | 0.5330 | 0.0220 | 20 |
| buttock | thickness_min | 25.9979 | -0.0220 | 0.3923 | 0.0141 | 54 |
| Abdomen | thickness_q25 | 42.4271 | -0.0402 | 0.4767 | 0.0285 | 20 |
| buttock | thickness_q25 | 47.7367 | -0.0402 | 0.4667 | 0.0102 | 54 |
| Abdomen | thickness_q75 | 63.1653 | -0.0487 | 0.7189 | 0.0074 | 20 |
| buttock | thickness_q75 | 67.6469 | 0.0090 | 0.9415 | 0.0001 | 54 |
| Abdomen | thickness_range | 114.2845 | -0.1763 | 0.7491 | 0.0058 | 20 |
| buttock | thickness_range | 211.9013 | -1.1368 | 0.2628 | 0.0241 | 54 |
| Abdomen | thickness_std | 23.2953 | -0.0403 | 0.7343 | 0.0066 | 20 |
| buttock | thickness_std | 47.5795 | -0.2868 | 0.2847 | 0.0220 | 54 |

bioRxiv preprint doi: https://doi.org/10.64898/2026.04.29.721704; this version posted May 4, 2026. The copyright holder for this preprint (which was not certified by peer review) is the author/funder, who has granted bioRxiv a license to display the preprint in perpetuity. It is made available under aCC-BY 4.0 International license.

## Supplemental Table 2. All IF-derived features

| feature | intercept | slope | p_value | r2 | n |
|---|---|---|---|---|---|
| ITGB4 Mean Intensity | 133.1 | -367.3 | <0.0001 | 0.396 | 60 |
| KI67 per unit Length | 176.6 | -0.0002 | 0.0283 | 0.094 | 51 |
| Solidity SD | 0.1384 | -0.0003 | 0.0020 | 0.224 | 40 |
| Solidity 25th percentile | 0.7191 | 0.0006 | 0.0086 | 0.168 | 40 |
| Solidity mean | 0.7976 | 0.0004 | 0.0130 | 0.152 | 40 |
| Minor Axis 50th percentile | 7.0575 | -0.0164 | 0.0173 | 0.140 | 40 |
| Minor Axis median | 7.0575 | -0.0164 | 0.0173 | 0.140 | 40 |
| Solidity 5th percentile | 0.5496 | 0.0008 | 0.0210 | 0.132 | 40 |
| Minor Axis mean | 8.5987 | -0.0182 | 0.0302 | 0.118 | 40 |
| Perimeter mean | 55.0466 | -0.1410 | 0.0603 | 0.090 | 40 |
| Perimeter 50th percentile | 33.0658 | -0.0554 | 0.0619 | 0.089 | 40 |
| Perimeter median | 33.0658 | -0.0554 | 0.0619 | 0.089 | 40 |
| Minor Axis 75th percentile | 9.8946 | -0.0211 | 0.0638 | 0.088 | 40 |
| Objects per mask area | 0.0002 | 0.0000 | 0.0824 | 0.077 | 40 |
| Perimeter 75th percentile | 59.1356 | -0.1477 | 0.0853 | 0.076 | 40 |
| Minor Axis 95th percentile | 19.1011 | -0.0500 | 0.1063 | 0.067 | 40 |
| Major Axis mean | 19.5406 | -0.0309 | 0.1308 | 0.059 | 40 |
| Solidity 50th percentile | 0.8295 | 0.0003 | 0.1390 | 0.057 | 40 |
| Solidity median | 0.8295 | 0.0003 | 0.1390 | 0.057 | 40 |
| Area 75th percentile | 130.1651 | -0.4012 | 0.1414 | 0.056 | 40 |
| Major Axis SD | 21.2797 | -0.0823 | 0.1637 | 0.050 | 40 |
| Area 5th percentile | 21.9088 | 0.0113 | 0.1816 | 0.046 | 40 |
| Minor Axis SD | 5.9095 | -0.0185 | 0.1839 | 0.046 | 40 |
| Perimeter 5th percentile | 15.6988 | 0.0078 | 0.1998 | 0.043 | 40 |
| Major Axis 75th percentile | 22.1019 | -0.0364 | 0.2072 | 0.042 | 40 |
| Minor Axis 5th percentile | 3.7134 | -0.0027 | 0.2109 | 0.041 | 40 |
| Perimeter 95th percentile | 152.5499 | -0.4154 | 0.2111 | 0.041 | 40 |
| Solidity 75th percentile | 0.8996 | 0.0002 | 0.2170 | 0.040 | 40 |
| Area 50th percentile | 61.0387 | -0.1293 | 0.2178 | 0.040 | 40 |
| Area median | 61.0387 | -0.1293 | 0.2178 | 0.040 | 40 |
| Perimeter 25th percentile | 21.5991 | -0.0133 | 0.2352 | 0.037 | 40 |
| Solidity 95th percentile | 0.9595 | 0.0001 | 0.2902 | 0.029 | 40 |
| Perimeter SD | 69.3208 | -0.2540 | 0.2972 | 0.029 | 40 |
| Major Axis 50th percentile | 13.2300 | -0.0096 | 0.3204 | 0.026 | 40 |
| Major Axis median | 13.2300 | -0.0096 | 0.3204 | 0.026 | 40 |

bioRxiv preprint doi: https://doi.org/10.64898/2026.04.29.721704; this version posted May 4, 2026. The copyright holder for this preprint (which was not certified by peer review) is the author/funder, who has granted bioRxiv a license to display the preprint in perpetuity. It is made available under aCC-BY 4.0 International license.

| | | | | | |
|---|---|---|---|---|---|
| Area mean | 119.8758 | -0.2574 | 0.3437 | 0.024 | 40 |
| Minor Axis 25th percentile | 4.8606 | -0.0030 | 0.3548 | 0.023 | 40 |
| Major Axis 95th percentile | 48.5337 | -0.0792 | 0.3700 | 0.021 | 40 |
| Area 25th percentile | 32.6197 | -0.0194 | 0.4903 | 0.013 | 40 |
| Major Axis 5th percentile | 6.6905 | 0.0018 | 0.5597 | 0.009 | 40 |
| Major Axis 25th percentile | 9.2552 | -0.0024 | 0.6376 | 0.006 | 40 |
| Area 95th percentile | 352.8748 | -0.4246 | 0.7048 | 0.004 | 40 |
| Area SD | 176.7203 | -0.1661 | 0.8549 | 0.001 | 40 |

bioRxiv preprint doi: https://doi.org/10.64898/2026.04.29.721704; this version posted May 4, 2026. The copyright holder for this preprint (which was not certified by peer review) is the author/funder, who has granted bioRxiv a license to display the preprint in perpetuity. It is made available under aCC-BY 4.0 International license.

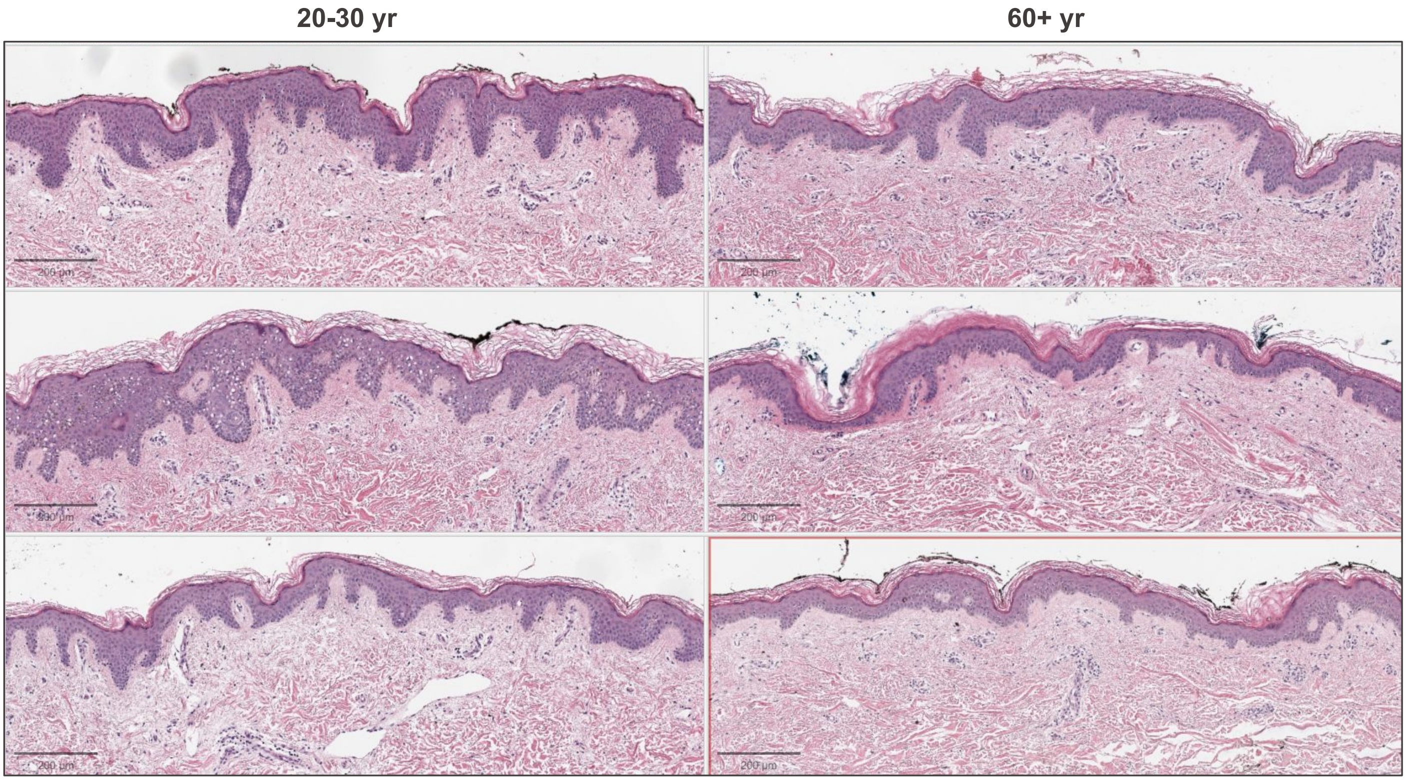


**Supplemental Figure 1. Representative images of young and old H&E-stained buttock skin.** Representative H&E-stained sections of paraffin-embedded skin tissue from young, middle-aged, and old groups.

bioRxiv preprint doi: https://doi.org/10.64898/2026.04.29.721704; this version posted May 4, 2026. The copyright holder for this preprint (which was not certified by peer review) is the author/funder, who has granted bioRxiv a license to display the preprint in perpetuity. It is made available under aCC-BY 4.0 International license.

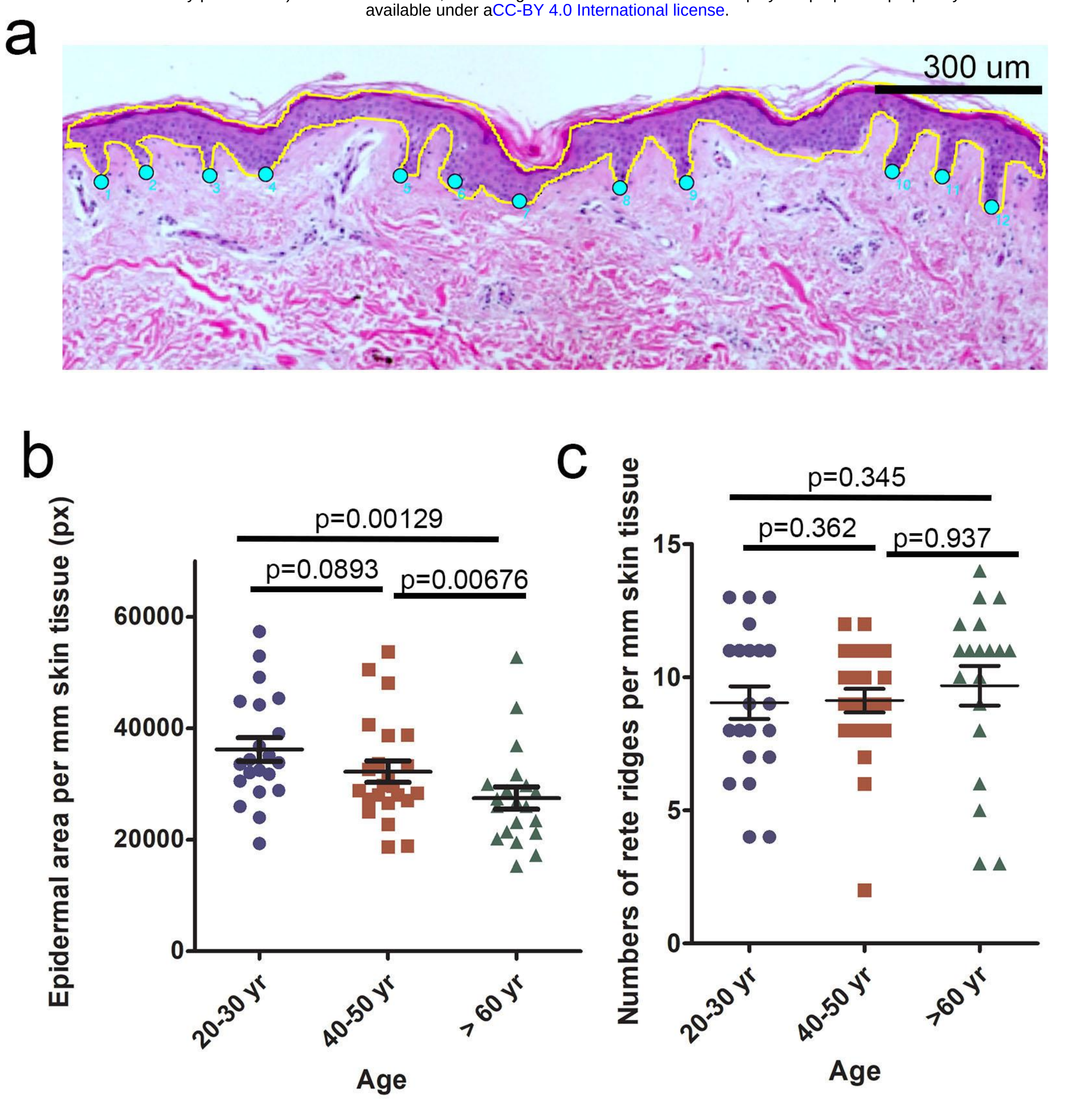


**Supplemental Figure 2. Manual quantification of epidermal thickness and rete ridge density.**
a. Representative H&E-stained sections of paraffin-embedded human skin tissue from young, middle-aged and old groups. The epidermal compartment was digitally delineated and measured, and rete ridges were identified as downward projections of the epidermis into the dermis. b. Quantification of epidermal area across age groups. The delineated epidermal region was measured using Fiji analysis software. Data are expressed as mean ± SEM (n = X per group, with Y fields analyzed per sample). Error bars represent SEM. Statistical comparisons among groups were performed using one-way ANOVA followed by Tukey's post hoc test. $p < 0.05$ was considered statistically significant. c. Quantification of rete ridge number across age groups. Rete ridges were annotated in each section and counted across predefined fields of view. Counts were averaged per sample to generate group means. Data are presented as mean ± SEM (n = X per group, Y fields analyzed per sample). Error bars represent SEM. Statistical comparisons among groups were performed using one-way ANOVA test. $p < 0.05$ was considered statistically significant.

bioRxiv preprint doi: https://doi.org/10.64898/2026.04.29.721704; this version posted May 4, 2026. The copyright holder for this preprint (which was not certified by peer review) is the author/funder, who has granted bioRxiv a license to display the preprint in perpetuity. It is made available under aCC-BY 4.0 International license.

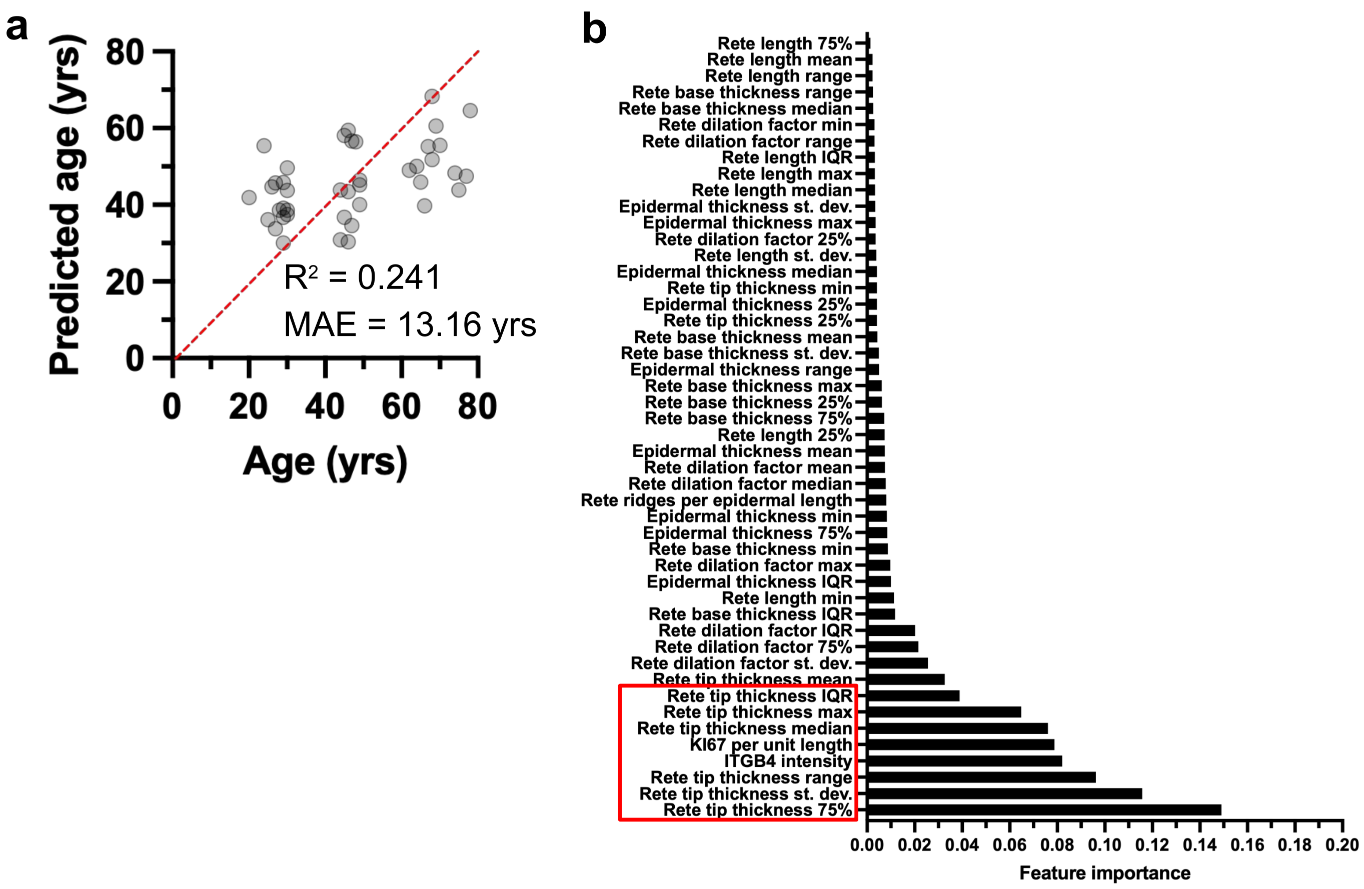


**Supplemental Figure 3. Full feature random forest regressor**

a. Actual versus predicted age as determined by a random forest regression model trained on the full feature set derived from the automated feature extraction methods. b. The full feature set derived from the automated feature extraction methods, ranked by the frequency it is chosen for splits that produce large reductions in impurity (error). The eight retained features used in the reduced polynomial random forest regression model are bounded in red.

bioRxiv preprint doi: https://doi.org/10.64898/2026.04.29.721704; this version posted May 4, 2026. The copyright holder for this preprint (which was not certified by peer review) is the author/funder, who has granted bioRxiv a license to display the preprint in perpetuity. It is made available under aCC-BY 4.0 International license.

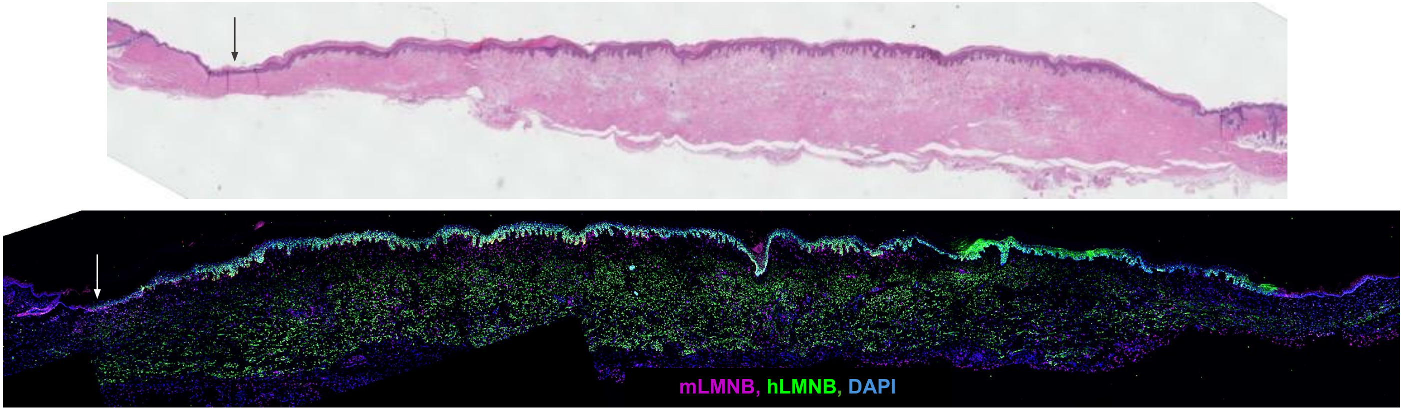


Arrows indicate junctions between the human graft and the host mouse skin

**Supplemental Figure 4. Example of a successful human skin xenograft**
Representative images showing successful engraftment of human skin as confirmed by H&E staining. Immunofluorescence staining with mouse- and human-specific LAMIN antibodies was performed to distinguish mouse and human cells within the grafted skin. Arrows indicate junctions between the human graft and the host mouse skin.

bioRxiv preprint doi: https://doi.org/10.64898/2026.04.29.721704; this version posted May 4, 2026. The copyright holder for this preprint (which was not certified by peer review) is the author/funder, who has granted bioRxiv a license to display the preprint in perpetuity. It is made available under aCC-BY 4.0 International license.

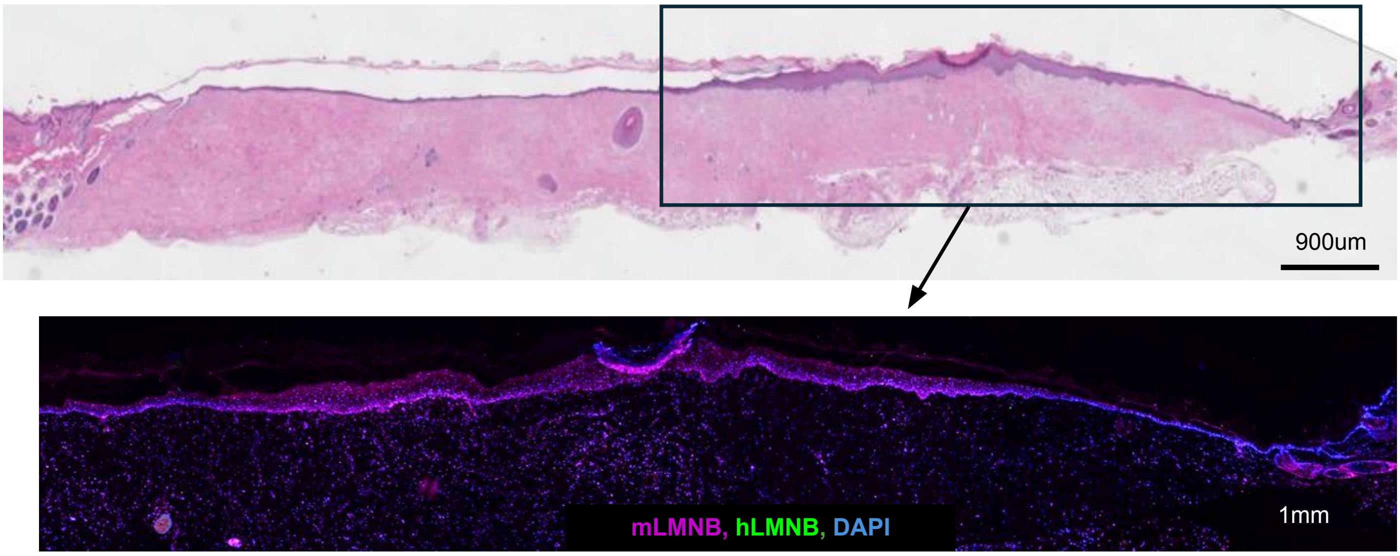


**Supplemental Figure 5. Example of a failed human skin xenograft**
Representative images showing failed human skin grafts. H&E staining demonstrates the absence of organized human epidermal structures, indicating graft failure. Immunofluorescence staining with mouse- and human-specific LAMIN antibodies was used to verify the cellular origin within the graft area.
